# A conceptualisation of scale-up and sustainability of social innovations in global health: a narrative review and integrative framework for action

**DOI:** 10.1080/16549716.2023.2230813

**Published:** 2023-07-17

**Authors:** Marietou Niang, Hassane Alami, Marie-Pierre Gagnon, Sophie Dupéré

**Affiliations:** a Department of Social Work and Psychosociology, Université du Québec à Rimouski, Lévis, QC, Canada; b Department of Health Management, Evaluation and Policy, School of Public Health, University of Montreal, Montreal, QC, Canada; c Faculty of Nursing Science, Université Laval, Québec, QC, Canada

**Keywords:** Scale-up_1_, sustainability_2_, social innovation, narrative review_3_, global health_4_, systems thinking_5_.hinking

## Abstract

**Background:**

The scale-up and sustainability of social innovations for health have received increased interest in global health research in recent years; however, these ambiguous concepts are poorly defined and insufficiently theorised and studied. Researchers, policymakers, and practitioners lack conceptual clarity and integrated frameworks for the scale-up and sustainability of global health innovations. Often, the frameworks developed are conceived in a linear and deterministic or consequentialist vision of the diffusion of innovations. This approach limits the consideration of complexity in scaling up and sustaining innovations.

**Objective:**

By using a systems theory lens and conducting a narrative review, this manuscript aims to produce an evidence-based integrative conceptual framework for the scale-up and sustainability of global health innovations.

**Method:**

We conducted a hermeneutic narrative review to synthetise different definitions of scale-up and sustainability to model an integrative definition of these concepts for global health. We have summarised the literature on the determinants that influence the conditions for innovation success or failure while noting the interconnections between internal and external innovation environments.

**Results:**

The internal innovation environment includes innovation characteristics (effectiveness and testability, monitoring and evaluation systems, simplification processes, resource requirements) and organisational characteristics (leadership and governance, organisational change, and organisational viability). The external innovation environment refers to receptive and transformative environments; the values, cultures, norms, and practices of individuals, communities, organisations, and systems; and other contextual characteristics relevant to innovation development.

**Conclusion:**

From these syntheses, we proposed an interconnected framework for action to better guide innovation researchers, practitioners, and policymakers in incorporating complexity and systemic interactions between internal and external innovation environments in global health.

## Background

Scale-up and sustainability innovation for health are ambiguous concepts, and little is known about what they mean and how their processes work in public health and, more broadly, global health. These concepts are poorly defined, under-theorised, and understudied [1–4]. This lack of precision in research has led to a deficiency in conceptual clarity [5,6]. Researchers, policymakers, practitioners, other stakeholders and even communities are encountering challenges in operationalising scale-up and sustainability in innovation development. These concepts’ qualitative and quantitative evaluation and systematisation remain challenging as they are often neither considered nor integrated into the design and implementation of global health projects. Every day, many global health innovations are implemented in lower-middle-income settings, but they face challenges in being sustained or scaled up from the pilot phase [7,8]. In these countries, the availability of upfront funding driven by donors typically determines the need to scale-up or sustain health innovations [9–12]. Donors generally have an accelerating effect on implementing and maintaining innovations during the often short periods when they have access to funding [13–15]. However, empirical evidence shows that most donor-funded and supported innovations in these countries fail to be sustained and scaled up in user organisations or communities and in the broader system [10,14–19].

Many conceptual frameworks used in global health aim to inform policymakers, donors, researchers, and implementers of best practices for scaling up and sustaining promising interventions. Nevertheless, they are normative or, in other words, standardised models and rules are proposed [17,20], with a focus on high-impact/immediate change [21]. Consequently, existing frameworks do not allow for a better understanding of how different functions and innovation processes interconnect and influence each other and what needs to be sustained in large-scale programmes and via which processes.

A paradigmatic orientation of global health studies and interventions in technology-driven innovation is driving this gap. This orientation emphasises products and processes [22], the passive and mechanistic diffusion of innovations [23], and instruments to stimulate economic growth [24,25]. This positivist paradigmatic orientation is limited to identify the sequences underlying innovation processes. Furthermore, frameworks developed in global health are uniquely related to scale-up components and underlying linear arrangements [26–28] and are less focused on sustainability [2]. To the best of our knowledge, no integrated frameworks enable policymakers and practitioners to scale-up and sustain innovations in global health. Thus, research needs to better understand the interdependence and interconnection between scale-up, sustainability, and overall innovation processes.

Recent frameworks have proposed an iterative and dynamic vision of scale-up linking with other innovation processes [11,28,29]; and have framed sustainability as a dynamic, emerging, and unpredictable process incorporating time and an evolving implementation context [2,30]. Notwithstanding, they still have a uniquely sequenced and deterministic/consequentialist conception of different scaling stages presupposing a linear development of innovation: ‘*Innovation is inherently good. So, we must spread its adoption*’ [31]. This consideration obscures the practices of adjustment, negotiations, alliances [23,32], efficiency [30], the arbitration of conflicts and power issues [32,33], learning, and even resilience [14], all of which are essential in innovation processes.

These frameworks have not highlighted the multiple interdependencies and contingencies between individuals/groups and innovation as well as between innovation and contextual systems. In this way, global health research has not been able to shed detailed light on the constraints posed by bureaucratic and symbolic cultures [34], socio-political reality [1,35], the effects of macro-contextual structures, such as the configuration of international health funding [36], and the dynamics of sanitary interventions at the micro level and health system dysfunctions [13,37] on innovation development. Given this, simply noting the multiple interactions, dynamics, tensions, and iterative nature inherent in innovation processes and contexts is no longer sufficient in research and practice. As recommended by some studies [38,39], researchers should embrace ‘fourth generation’ approaches that are complex, recursive, ecological, and critical to better understand innovation processes, conceive frameworks, and generate empirical evidence.

This narrative review presents the state of knowledge on the processes of scaling up and sustaining social innovation for health and produces an evidence-based integrative framework of these processes in global health. This review has a broader scope than what we present in this manuscript. Other results have already been published [40,41].

## Method

### Purpose and theoretical perspective of the narrative review

To address the limitations observed in the scientific literature, we conducted a hermeneutic narrative review to better understand the scale-up and sustainability processes of innovation in global health. This type of review is characterised by its inherently interpretive, critical, and inductive process [42] in which the researcher ‘*engages in an ever-expanding and deepening understanding of a relevant body of literature*’ [43]. Our goal was to thoroughly understand the scientific literature around the scale-up and sustainability of global health innovations and to propose new conceptualisations of these ideas and their processes in global health. This narrative review was designed first by changing our view of global health innovation, thus suggesting consideration of social innovation for health instead of the vague, broad concept of health innovation. Second, we conceived this narrative review using the science of systems thinking approach. This approach allows us to assess the importance of considering the complexity of the research object. In this approach, recognising the coevolution between the innovation (as a system) and its environment is essential to capture how innovation processes occur and evolve in time and space [44,45]. Adopting the *systems thinking* approach facilitates the researcher’s broader vision of innovation processes as recursive and self-organised systems [44,46].

### Social innovation for health: definition

The concept of health innovation is vast. Most studies focus on it from a narrow technical-economic perspective that promotes efficiency and cost-effectiveness, encouraging the implementation of medical, technical, or economic innovations to the detriment of other innovations [40,41]. In this review, we position health innovation within the social paradigm, specifically social innovation for health, an emerging concept in public and global health [7,47]. In this manuscript, social innovation for health is understood as a type of innovation that focuses on interactions, dynamics, and synergies between different actors and contexts to address health problems, improve living conditions, and proposes lasting social change and transformations in complex adaptive systems [47,48]. Social innovation is a multidimensional concept that incorporates from different theoretical perspectives and disciplinary traditions [47]. However, as in another study [49], we do not restrict our conception of social innovation to the bottom-up process vision or innovation initiated solely by the community. Instead, we are committed to a perspective of social innovation in health that emerges from communities, institutions, health systems, profit or non-profit organisations, and partnerships between these actors. In global health, donors and public health experts from high-income settings often initiate and support innovations. Generally, the beneficiaries of these interventions in lower-middle-income settings are separate from their design, implementation, scaling up, and sustainability. Given this state of affairs, it is essential to pay particular attention to the balance of power dynamics between different stakeholders and the combination of various environmental, economic, ideological, social, religious, historical, and cultural issues to address contemporary societal challenges, including social and environmental inequalities [40,50]. Social innovation differs from the techno-economic perspective of innovation in that it uses democratic processes that promote the creation of sustainable social value from a perspective of equity and social justice as well as epistemic and ontological justice. For these types of innovations to bring about essential changes in the setting or population of adoption, it is crucial that they benefit the people who need them and that they are sustainable for organisations and institutions. It is, therefore, essential to better understand the conditions that either promote or hinder the scaling up and sustainability of social innovations in health. However, implementers and stakeholders need to closely study and be able to recognise social innovations [51].

#### Research and acquisition of the literature

**Figure 1.**
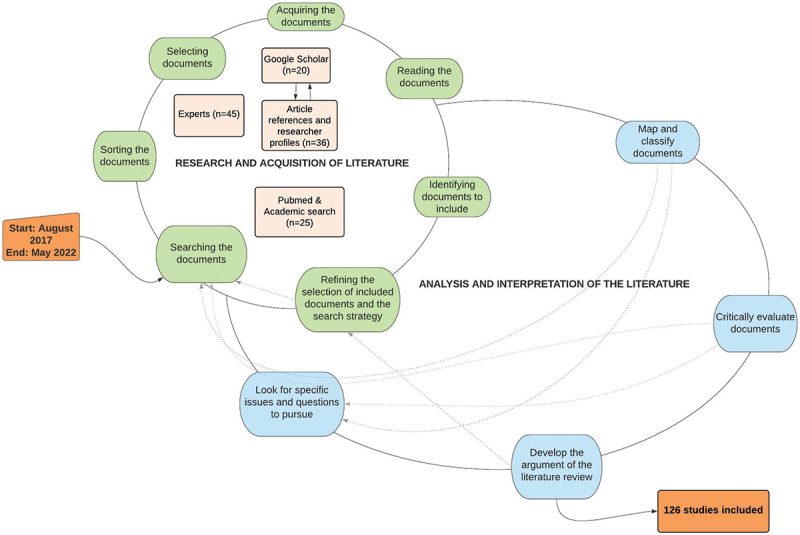
Process of searching, acquiring and interpreting the literature adapted to [43].

To collect, analyse, and interpret the literature, we used a framework that includes two interlocking and recursive hermeneutic circles (Figure 1): (1) the circle of research and study acquisition and (2) the circle of study analysis and interpretation [43].

We conducted the research and literature acquisition processes in three iterative stages. Firstly, we consulted some experts in the field of public and community health, who suggested about 100 documents. We selected 45 relevant documents, which are included in this review. This stage allowed us to develop our definitions and understanding of scaling up and sustainability. Also, without a clear purpose and description of social innovation in the scientific literature, we have included any innovation with technical, organisational, social, and political combinations or that proposed social change through a participatory and non-linear process. Second, we conducted new research on the Google Scholar database and listed 20 pertinent documents. Then, as our understanding of the study concepts deepened, further research was done using the references of relevant articles and the Google Scholar profiles of some researchers to identify other studies. We found 36 additional empirical, philosophical, and theoretical studies in this search in several disciplines. We did not delineate the time window for these two stages. Finally, given the need to contextualise this review in global health, we conducted new research on the PubMed and Academic search databases (*n* = 3673 documents). We included 126 documents overall in this review. This research was limited to a time window between 2000 and 2020. The search and acquisition of the literature stopped when new studies needed to provide further information to clarify the review’s objectives or support the development of the argument. The keywords used in our research are presented in Table 1.

**Table 1.** Overview of keywords used in searching and acquiring literature.

Social innovation	‘Social Innovation*’ OR ‘Social Change’ OR ‘Community based intervention’
Scale-up	(‘Scale up’ OR Scal* up) OR ‘scaling up’ OR ‘scale’ OR ‘Diffusion of Innovation’
Sustainability	**‘Sustainability’ OR ‘Confirmation’ ‘continuation’ OR ‘Durability’ OR ‘Incorporation’ OR ‘Institutionalization’ OR ‘Routinization’ OR ‘Stabilization’**
Global health	‘Global health’ OR ‘Public Health’ OR (‘Developing countries or developing nations or third world or low-income countries’)

#### Analysis and interpretation of studies: a systems thinking approach

We have used systems thinking to analyse and interpret the data from the literature. This approach enabled us to go beyond the primary objective of literature reviews to summarise and synthesise scientific literature results. We are going beyond simple considerations of the components of innovation or the contexts identified in the literature. We are highlighting the interconnections between different elements, particularly the effects of these interdependencies in scaling up and sustaining health innovations. Finally, we propose a conceptual framework for future studies.

## Results

The analysis and interpretation of the studies made it possible to systematise definitions of scale-up and sustainability and several determinants that influence the conditions for the success or failure of scaling up and sustaining social innovations in health.

### Scale-up and sustainability definitions and conceptualisation

Regarding the literature, scale-up can be conceptualised into three complementary dimensions: quantitative, qualitative, and political. The quantitative dimension focuses on geographic expansion to increase the inputs needed, such as financial, human, or capital resources [4,52]. The qualitative dimension refers to equitably and sustainably extending innovation’s impact. Indeed, scale-up goes beyond simply replicating an innovation; it concerns disseminating knowledge, processes, and technologies [53] while paying close attention to hard-to-reach populations to ensure equity and inclusion. It is then multidimensional and occurs in complex social, political, institutional, cultural, and economic contexts [26]. To this end, some authors viewed scale-up as a collective, iterative, and interactive learning process [53] or a non-linear and emerging process that allows for the influence of cultural or social beliefs and norms (scaling deep) [54]. In this dimension, we must also consider the increase in the number and type of innovation activities to ensure the sustainability of the impact; this was conceptualised by Uvin [52] as a functional scale-up component. The policy dimension refers to the specific objective of integrating and/or institutionalising a proven innovation into the existing health system while focusing on its institutional capacity building and sustainability [26,55,56]. This taxonomy should not be taken for granted; it involves a consideration of the ways and values underlying scaling up. Thus, unlike the private domain, scaling up social innovations should not focus on organisational growth but rather on expanding social impacts so that the innovation can meet the needs of all users. In this sense, scaling up implies sense-making and adaptation [57], the renegotiation of ‘negotiated orders’ [33,58], reinvention processes [59], conflict arbitration [32], and changes in political, legal, and cultural norms or simply in power relations and dynamics [54,60].

**Figure 2.**
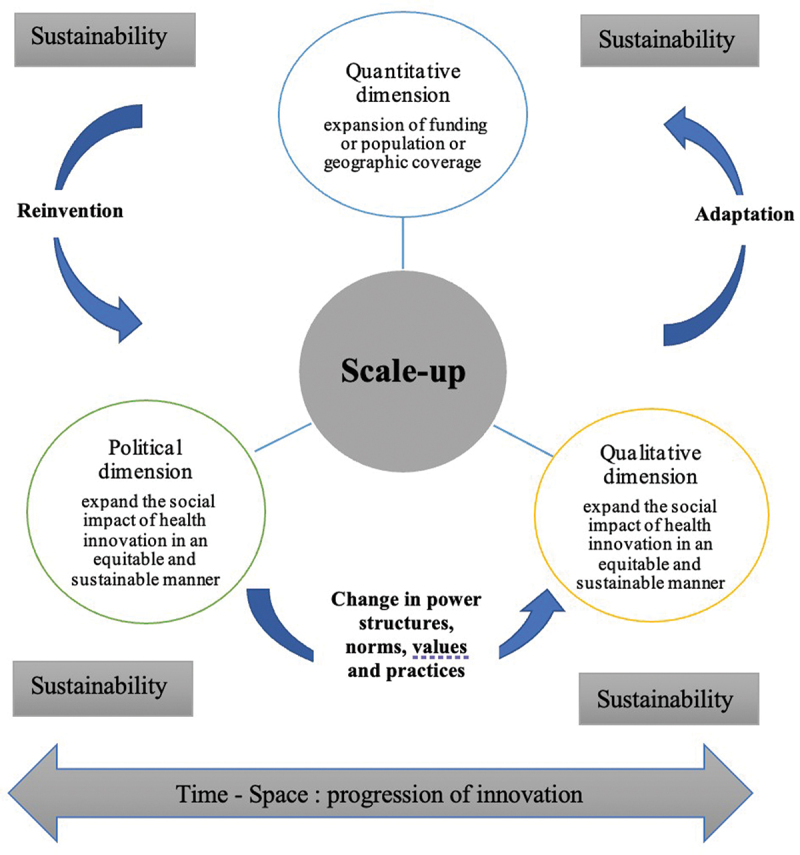
Scale-up and sustainability definitions and conceptualisations.

Notwithstanding, few studies address the processes of scaling up and sustainability concomitantly. For example, in the systematic review on scale-up public health interventions in low- and middle-income countries [1], out of 27 studies included, only 11 defined scaling up; of these 11, seven highlighted sustainability. This finding shows the existing dichotomous vision of these innovation processes and the lack of clarity on what scale-up and sustainability mean, especially in global health. In the scientific literature, there is often an implication of complementarity or similarity between scale-up and sustainability. On the one hand, several authors point out that scale-up requires enhancing and paying particular attention to the sustainability of the extension of social impact over time [26,61]. Sustainability is often seen as a process that occurs over the temporal progression of innovation to better capture variations in the innovation process [62]. However, the spatial progression of the innovation is also relevant to sustainability. For example, one study [17], while drawing on the experience of non-governmental organisations (NGOs) with free healthcare Burkina Faso, Mali, and Niger, highlights two sustainability processes: one at the local level and one at a national level. These two processes show that sustainability begins at the pilot project level, where NGOs are often responsible for maintaining innovation. In expanding the geographic coverage or impact of the innovation, the innovation’s implementation and continuation are the health institutions’ responsibility. This approach leads the authors to conclude that the innovation may be successful at the local level in the short term, yet its sustainability at both the local and national levels has been a long-term failure. In this sense, it is crucial to consider sustainability as being transversal in a scaling-up process; it is necessary to distinguish the sustainability of the innovation (its goals, scope, structure, and components) from the sustainability of the scaling up [14]. This observation leads to considering in Figure 2, on the one hand, the importance of studying sustainability processes and scaling up as being interconnected and interdependent. On the other hand, sustainability is a phenomenon that manifests itself in time and space according to the evolution of the innovation and/or context.

A wide range of research focuses solely on the sustainability of innovations. In this research, sustainability has been studied from four different theoretical perspectives according to a systematic review [63]. The diffusionist approach is mostly used in research and implementation in global health, and sustainability is considered as the last stage of the innovation life cycle [64,65]. In this model, innovation development follows sequential/deterministic and linear phases: ideation, initiation, development, adoption, implementation, sustainability (or discontinuity), and scale-up [66]. Innovation perpetuates when all the preceding stages have been successful. It does not allow innovation sustainability to be a recursive, iterative, reflexive, and learning process [64,67].

In contrast to this linear vision, other studies rooted in complexity theories, including the systems approach, propose that sustainability is a non-linear process that emphasises change, equilibration, adaptation, and recursion capabilities of innovation that coevolves with its environment [2,68,69]. Another non-linear approach is the ecological theory, which proposes that sustainability is a multidimensional, dynamic, and evolving process during implementation [30,67]. In a dynamic and emerging vision, Chambers et al. [30] suggest that the context of the innovation, being in continuous change and sometimes in unexpected ways, considerably influences the sustainability process. Adaptation is then inevitable and desired and must be accompanied by processual and continuous evaluations, especially since there is feedback between sustainability and other innovation processes [14].

### Scale-up and sustainability of social innovation for health determinants

To make the complexity of the eclectic data intelligible, we organised this part of the results into two main subsections: (1) the innovation’s internal environment and (2) the innovation’s external environment. The internal environment concerns the various elements specific to innovation: the characteristics of the innovation, notably its structure, and the organisational characteristics of the organisations that initiate or support the innovation. The external environment refers to any element or context that can interact with the innovation. This data organisation allows better highlighting of the themes emerging from the interpretation of the studies while not losing the vision of the existing interconnections, interdependencies and even contingencies between the internal and external environments of innovation.

#### Innovation’s internal environment

##### Innovation characteristics

###### Effectiveness and testability

Effectiveness and testability are prerequisites for the scale-up [1,26,51,70,71] and sustaining of a health innovation [62,64,72,73]. The innovation must then be tested through methodologically robust research (qualitative and quantitative), such as randomised controlled trials. This approach has been proven to be cost-effective and feasible [27] and is used to inform policymakers and other funding or technical partners of the importance of the innovation [74]. The testing stage of the innovation to be scaled is a crucial opportunity to firm up the conviction and willingness of policymakers, leaders, and frontline staff to support change [28]. For example, the national policy Community-based Health Planning and Services in Ghana was designed based on scientific evidence from an experimental trial of the Navrongo Project and its first replication site in the Nkwanta District [75]. The pilot project sites served as social learning environments for national policy implementers [75]. Furthermore, innovation effectiveness in the pilot sites may decline during scaling [76]. Another study found that large-scale obesity prevention and treatment interventions showed less than 75% of the effect size established during their efficacy trials [8]. Researchers recommended designing scalable innovations and determining an acceptable degree of decline in effectiveness [76] as well as evaluating and changing approaches during the scaling process [77].

####### Monitoring and evaluation system

Evidence supports that the research and implementation of a monitoring and evaluation system throughout the innovation life cycle are success factors for scaling innovation [26,78,79]. Studies focusing on sustainability have also indicated that evaluative research on innovation effectiveness should not be done statically or only at the initiation phase of the innovation. They should be cyclical and co-evolving with the innovation process to allow for learning and adjustments to ensure innovation sustainability [2,30,33,64] and to assess whether to keep or change its design and/or scope [80]. In addition, the effectiveness of community-based innovations is often more challenging to evaluate in terms of evidence. This is often due to inappropriate evaluation methods that focus on performance outcomes rather than processes or use solely quantitative measures and therefore fail to capture the systemic context and social impact of these types of interventions [33,81] or the dynamics of social change [82]. Considering the dynamic and evolving nature of innovation during scaling up, further methods aside from the randomised controls commonly solely are required to evaluate global health interventions’ large-scale impact required [3,33,81,83]. It is essential to combine different sources of evidence and develop new models that integrate plausibility, appropriateness, and complex approaches [81]. The reputation of an innovation as being ‘effective’ in the community, even if not objectively proven, can be a lever for sustainability [73]. Indeed, beneficiaries could quickly adopt and support the innovation, especially in the early stages of its scale-up, as observed with the seasonal malaria chemoprevention innovation in Burkina Faso [14].

Furthermore, Hage and Valadez [84] noted that in the scientific literature, institutionalisation and sustainability are often measured or understood through the ‘stability’ of norms or practices. However, these authors emphasise the importance of incorporating the notion of change, focusing on continuous programme improvement using data from programme evaluations and monitoring. In moving from the idea of stability to social change to study institutionalisation and sustainability, these authors emphasise the capacity to learn (through continuous training and experience) and the use of evaluation data as critical events. There are also community monitoring tools for evaluating the social innovation process. For example, in the Community and District Empowerment for Scale-up project for child survival in Uganda, community dialogues between health professionals and community leaders were instituted [85]. A post-dialogue monitoring system assessed the process and effects of these community dialogues through indicators of dialogue participants’ attendance and activities, community contracts generated during each dialogue, adding contextual information, and making plausibility arguments about observed effects [85].

###### Simplification process

The simplicity of the innovation concerning the hardware (e.g. technique) and software (e.g. user guides) has been identified as a significant predictor of successful scaling [12,27,71,86–89] and sustainability [26,72]. Understanding the degree of complexity of an innovation seems essential to identifying strategies for overcoming implementation, scaling-up, and sustainability constraints [87]. Some authors argue that the complexity of an intervention can be understood by taking into account the number of components in the intervention; interactions between intervention components or interactions between the intervention and its context, or both; and the broader system within which the intervention is introduced [90]. The simplification process can then reduce the innovation’s technical complexity through various means, such as standardising and simplifying procedures and delegating tasks that often require changes in policies, regulations, or laws [26,27,74,87]. A study of antiretroviral therapy for people living with HIV in South Africa found that the flexibility of the intervention, person-centred care, and the availability to patients through various mechanisms were the main factors that contributed to the programme’s success [91]. In global health, simple, low-cost, evidence-based innovations, such as low-cost, effective vaccines or drugs, will be easier to promote to policymakers [92]. Thus, policymakers are more likely to support technological innovations or emphasise the technical nature of an innovation because they are simpler to understand and more widespread than organisational or social innovations [93].

Edwards’ review [94] of the literature found that discrete innovations (such as vaccines or antiretrovirals for HIV) are considered more likely to be scaled-up because of their demonstrated effectiveness and efficiency in delivery. However, despite the simplistic nature of discrete innovations, there are complex causal pathways to consider when scaling them up [56]. Edwards suggests that effectiveness studies must consider the complexity of the interface between innovation and systems adoption. Apart from discrete innovations, this author distinguishes two much more complex innovation types: multi-component or multi-level innovations and paradigmatic innovations. Scaling-up and sustaining these complex interventions requires a coherent and synergic approach involving multiple sectors beyond health and the consideration of relationships and interactions, even contingencies, among the elements of the innovation and contextual factors at different levels of the system. These include organisational policies, legislation, regulations, community and political commitments, leadership, and demand for services. However, the complexity issue should not only be thought of as related to the structure of the innovation, as it can also emerge during the process of implementing and scaling-up the innovation. For example, one study on membership clubs that provide differentiated care to HIV-positive people in South Africa found that in its pilot phase, the innovation was simple with a unitary form of management and governance [95]. After a decade of existence and scaling-up, the innovation has become more complex with different components of care, management, and governance at different health system levels.

###### Resources required

Financial, material, and human resources are necessary for health innovation scale-up and sustainability processes. In developing countries, issues with non-financial resources, such as human resource shortages or logistical challenges, are one of the potential constraints to scale-up and sustainability [12,87,96,97]. A study of the introduction of a mobile health intervention (RapidSMS) in Malawi and Zambia found that the most significant challenges to scale-up and sustainability are related to a lack of skilled technical staff to maintain services and databases [96]. Another study in Indonesia indicated that a lack of resources for training and equipment and gaps in provider skills constrained the success of the scale-up of community-based neonatal resuscitation [98]. However, financial resources remain an essential determinant for the sustainability and scaling of health innovations and money, or a lack thereof, which is often cited in studies as a fundamental barrier to the success and continued operation of an innovation [1,2,64,72,99]. With this momentum, it is important to consider training as a fundamental element to facilitate the scaling up of innovations [1].

On the other hand, training is not only a means of promoting the expansion of innovations, as it also makes it possible to improve practices and helps ensure the sustainability of the various actions undertaken. Training during follow-up activities facilitates the adaptation of the transmission of knowledge to the realities of the environments [15,100]. In this sense, the continuous training of human resources involved in the innovation, such as health personnel, policymakers, or personnel of user organisations and communities, is necessary during the scale-up process to sustain knowledge translation.

##### Organisational characteristics

###### Leadership and governance

Strong leadership and governance skills facilitate innovation’s scaling-up and sustainability [71,72,101]. On this point, Yamey [71] argues that leadership and governance are essential characteristics for those responsible for implementing an innovation and are critical to successful scale-up. The involvement of the implementers, other state and non-state decision-makers, and leaders at different levels of the health system facilitate scaling up [95]. Indeed, Johnson et al. [72] noted in their literature review that leaders or champions can be found inside or outside of the system, and they promote innovation to facilitate the process of sustainability. Identifying champions is undoubtedly a good strategy for scaling up; still, it does not always guarantee the sustainability of an innovation or scaling up, especially in contexts where there is a change in the actors involved in a pilot project [14]. Also, the literature review by Stirman et al. [62] showed that in a sustainability process, ‘effective’ leaders, who encourage negotiation, relationships, transparency, trust, and shared decision-making among different stakeholders, can sustain and nurture new ideas or practices.

###### Organisational change

Organisations’ structural and cultural characteristics significantly influence stakeholder adoption of an innovation and its success [23]. Simmons et al. [26] identified two types of organisations that can support the scaling up and sustainability of innovations: (1) user organisations that adopt and apply the innovation and (2) support teams that promote the scaling up of the innovation. The relationship between these two organisational entities is highly dynamic. Collaboration and partnership between the support team and the user organisation is one of the most critical elements of the scaling up of an innovation [98]. In a scaling-up process, the user organisation becomes the support team, which is conducive to successfully scaling up the innovation [98]. Introducing innovation into an organisation is not only about keeping up with technological change but also about promoting organisational change. Therefore, it is essential for change agents to ensure that organisational change matches existing organisational strengths and identify strategies or areas for directing change efforts [102]. It is important to note that the innovation must be compatible and aligned with the practices and goals of the user organisation to facilitate scaling up and sustainability [26]. According to Cooley and Kohl [27], in a scaling-up process, the characteristics of the user organisation that initially adopted and implemented the pilot project can be retained, recreated, or substituted to ensure the successful scaling up of the innovation. According to these authors, taking an interest in the user organisation’s readiness, culture, values and principles, skills, objectives, and capacities regarding resources, leadership, management, and internal and external collaboration is essential. Monitoring and evaluating these factors are equally vital. To do this, the administrative or organisational units responsible for monitoring, integrating, and using the innovation must have the organisational capacity for scaling-up and sustainability. These organisational capacities refer to the adequacy of inputs, that is, knowledge, financial resources, trained and skilled personnel, the necessary room for manoeuvring, and strategic partnerships [103]. Meanwhile, the outputs refer to the capacity to deliver quality services to achieve innovation objectives concerning the expected results for the innovation’s users [103].

###### Organisational viability

Organisational viability refers to the financial dimension and other supports and relationships an organisation needs to pursue its objectives [104]. In global health, international donors generally fund innovation; this can compromise the organisational viability of the user organisation (NGO or government) and, consequently, the sustainability of the innovation [14,17]. It is essential to safeguard the autonomy of the organisational user by ensuring their integration into a social network that allows them to access knowledge and other types of resources [104]. Finally, to ensure the maintenance and continuity of the innovation, some authors recommend that the administrative or organisational units responsible for carrying out the innovation’s activities should adopt participatory, inclusive, transparent, and adaptive governance that allows for the involvement of all stakeholders in decision-making, innovation modelling, and other activities [14,105]. However, in global health, many organisations with divergent interests and agendas, even if they are involved in scaling up and sustaining processes. Some studies have noted the existence of certain preferences and the pursuit of divergent purposes, especially between state, local and international NGOs [10,16,106]. Furthermore, actors from the Global North and health experts often have control over resources and the decision-making process, while users from recipient in the Global South do not participate in formulating innovative solutions (‘subordination relationship’) [15,18,100,106,107]. Nevertheless, few empirical studies have shed light on power dynamics and how they operate in global health interventions. In this vein, the idea that global health interventions are ‘apolitical and neutral’ is still naively widespread. Some experts have highlighted the importance of studying the functioning of power in global health interventions [107,108].

#### Innovation’s external environment

##### Receptive and transformative environments

For many authors, scaling-up and sustaining innovation cannot occur in a non-receptive environment [2,28,64,109,110]. Implementers must often adapt an innovation to social, cultural, political, economic, and institutional contexts [4,70,95]. It is essential to consider the heterogeneity of the population and context and the difference between the pilot site and scaling-up site [109,111]. Implementation contexts are heterogeneous, complex, dynamic, emerging, and continuously changing [112]. Considering this, adapting an innovation requires changing the traditional linear vision, which focuses on testing innovations’ effectiveness, efficacy, and fidelity, as the first step in innovation [30,109]. To overcome the tension between fidelity and adaptation, one avenue to explore is the design of adjustable interventions; this would allow the essential ideas of the initial project to be preserved and ensure the project is properly suited to the realities of the environment [113]. Developing a culture of adaptation, flexibility, humility, and openness to change is the key to scaling-up success [77,114] while focusing on sustainability [26].

##### Values and norms compatibility

Innovations that are more compatible with social, even moral, values and norms are more likely to be sustainable [1,2]. Implementers can adapt innovations to political, programmatic, economic, social, historical, and cultural contexts [2,26]. To foster a responsive environment, addressing existing power structures and dynamics (e.g. symbolic and structural violence, colonial trauma) in communities is essential to carrying out targeted actions with disadvantaged, poor, and marginalised people and groups. In this sense, after studying two community-based programmes to empower poor and marginalised women in India and South Africa, one study [110] proposes developing transformative communication to address these power structures and dynamics. It is also necessary to ensure that they have access and the capabilities (and the necessary freedom) to use and mobilise symbolic and material resources as well as opportunities to practice their agency. In global health, donors’ values are essential for innovation development. A literature review [21] noted that in developing countries, technical partners and donors can promote quality improvement models developed in Western countries. These approaches pose many challenges to the long-term sustainability of interventions because of their lack of coherence and contextualisation with local systems, resources, cultures, values, and even history. These authors recommended that incremental and context-specific improvements be emphasied over predefined models or methods.

Nevertheless, donors’ provision of external resources can adversely affect local governments’ capacity for macroeconomic management, planning, budgeting, and service delivery. Furthermore, aid dependence undermines the commitment of the local government to implement not only necessary reforms and local governance mechanisms [4,115] but also to implement more comprehensive strategies, for example, strategies that relate to sexual and reproductive rights [116]. Depending on donors’ conditionalities, countries or organisations challenge contextualising the intervention or considering the population’s needs during innovation implementation.

##### Community and individual levels

At this level, some determinants that constrain the scale-up and sustainability of innovations include a lack of community mobilisation, insufficient advocacy efforts in communities, a lack of community organisations or structures, and failures in service demand [117,118]. A literature review on innovation sustainability in sub-Saharan Africa found that community ownership and mobilisation facilitate an intervention’s sustainability [2]. The effective participation of stakeholders and developing their sense of belonging to an innovation can contribute to sustainability and scale-up at the beginning and end of implementation. The innovation’s accessibility to the target populations is essential to facilitate scale-up, considering that supply and demand are intrinsically linked [94]. Specifically, for some demand-side barriers, it has been recommended in the literature to use community health workers to deliver services, employ outreach strategies, and promote community participation in the planning and implementation of innovations [71,119]. If health districts can build innovations from and with existing communities’ resources and structures, it could facilitate their survival, even after external funding and technical support [2,120].

##### Health system and organisation levels

At this level, several constraints can arise in scaling up and sustainability processes, such as shortages and the inequitable distribution of skilled personnel, weak management capacity and technical knowledge, inadequate supervision, a lack of infrastructure and equipment, inadequate medical provision, inaccessibility of health services, and a lack of intersectoral action and partnerships for health between government and civil society [121]. The lack of a clear strategic vision and alignment, or even hostility or contradictory political decisions in the health system, can hinder the development of innovations [23]. However, decision-makers or implementers could mitigate some of these constraints by addressing systemic problems, including resource management and planning [4]. These systemic problems are related to the organisational and bureaucratic culture that often impedes innovations’ scaling up and sustainability [17]. In this sense, consideration of health care workers and users in a health system is essential [101].

##### Other contextual characteristics

These characteristics, such as levels of education, national stability, corruption, poor governance of health systems, the many interfaces between global health projects and local institutions, and the physical, climatic, and geographic environment, are relevant in the innovation process [15,121,122]. In addition, gender inequalities and cultural norms and practices are also contextual factors that are crucial determinants in scaling up and sustainability processes [123]. In this sense, it remains essential in both research and interventions in global health not to consider marginalised people as a homogeneous group and not to invisibilise existing territorial inequalities. For example, a study on HIV in Tanzania found that women living in urban areas tended to be better at attending and applying preventive education than women living in rural areas [124]. This study suggests that this finding is related to rural women’s lower capacity, resources, power, and knowledge of how to access maternal and reproductive services and resources compared to urban women. Therefore, this author recommends targeting these different groups of women for better acceptability and dissemination of innovations.

## Discussion

We subdivided this discussion section into three parts. The first part concerns the limitations and strengths of the narrative review and general learning about the concepts studied. The second part presents observations and analyses we have made of the empirical results of the review. In the third part, based on our analysis of the empirical results, we propose an integrative conceptual framework for scaling up and sustainability that would allow us to improve on the shortcomings observed in the literature.

### Limitations and strengths of narrative review: learning about concepts

This narrative review has taken on the challenge of analysing a wide range of publications on the determinants of sustainability and scaling up in global health. By using this method, this review could go beyond the influence of the characteristics of the innovations and their implementation contexts. It has succeeded in showing ‘all’ the interactions between different determinants of innovation and the interdependent nature of the scaling-up and sustainability processes. However, we noted some limitations that are not related to the procedures of narrative research but rather to the **sparse** nature of the literature analysed. Many studies generally focused on a single concept of scaling up or sustainability, but only a few case studies examined both concepts [16]. Our analysis has limitations in linking studies. Researchers often used different definitions, concepts, or methods for scaling up, sustainability, or social innovation. These concepts often refer to either a specific outcome, process, both, or a method (participatory research).

In consequence, it was a challenge to make meaningful comparisons between different studies. Most studies did not use the term ‘social innovation’ to refer to the type of innovation being studied. As a result, the studies were analysed based on the definitional and conceptual elements identified for social innovation at the beginning of this manuscript.

Nevertheless, the narrative review’s iterative method helped overcome these challenges. First, to facilitate our understanding of the concepts studied, we schematised the critical elements to consider in scaling and sustainability’s definition (Figure 2). This figure helped us to situate the authors’ perspectives and to deal with the need for more consensus with these notions in this review. It could be a step forward in research and practice to better conceptualise and operationalise these concepts in global health.

Second, the depth of analysis and interpretation of the literature revealed that scaling up and sustainability often share the same determinants, facilitating the aggregation of essential elements identified in the studies. Also, through the opportunity to critically analyse the literature and search for specific issues or questions to be explored in the narrative review, it was possible to identify relationships and interconnections between different themes or arguments. This review could be very useful even in lower-, middle- and higher-income settings where the determinants that emerged from this review explain the failures or successes of several innovations.

### Analysis of empirical results

We observed some limits in the literature reviewed in the determinants of scaling up and sustainability of social innovation, particularly in global health. In existing studies, little importance is placed on the relationships between the processes of innovation, scaling up, and sustainability. For example, innovation promoters, such as researchers and implementers, often consider scale-up and sustainability separately or interchangeably, and are not related to the innovation’s whole process, context, or history. Studies considered few factors or substantive elements that could explain the success or failure of the scaling up or sustainability processes. In addition, the interactions or feedback loops between innovation processes and the context in which innovations are implemented must be clarified.

Moreover, the literature has noted that few studies understand the processes through which innovations are (re)negotiated, (re)defined, adopted, implemented, and supported, particularly when scaling-up is not done systematically or following long-term planning [125]. On this point, although the framework of Greenhalgh et al. [23] tried to show the inherent complexity of innovation processes, it nevertheless does not provide a definitive answer, as indicated in their study. These authors raised several shortcomings that still exist in the study of innovations. For example, the implementation and diffusion processes of complex innovations are not well considered as well as the dynamic nature of the interactions of their determinants and the context [39]. We propose studying innovation processes by considering components and events as an integrated whole. In this way, we must study and implement innovation processes from a systems and processual perspective to better understand innovation’s evolution dynamic, changing contexts and socio-political complexity.

Based on this review, it remains essential to study scale-up and sustainability processes to pay close attention to the values that innovations encompass and symbolise and those of the actors and organisations involved. Often, in studies of innovation processes, the issue of values is poorly elucidated in the public and community health literature [126]. In Greenhalgh et al.’s study [39], values are equated with the notion of the relative advantage developed by Rogers [127]. We consider that these two notions are different. Relative advantage refers to the degree to which potential users believe or perceive the innovation as representing an improvement [127]. This often relates to moral considerations or purposes driven by users’ value systems and involves the user’s preferences, ability, and freedom to choose based on specific purposes, such as economic, social, or preference reasons [127]. However, from our point of view, the relative advantage is insufficient to show the full ontological scope value. Indeed, values are more encompassing. While abstract, it allows us to understand the intelligibility or rationalities of the actions undertaken in an innovation that can explain their potential for success or failure. In this sense, values have the function of stabilising innovation, but they also participate in defining behaviours or states through time and space. A study by Niang [14] comparing three innovations observes that the values given to an innovation are different according to the actors and organisations who implement or lead it. This study reveals that innovations supported and recommended by the scientific and international community focus more on effectiveness, cost, and rentability. However, innovations initiated and supported by communities promote the perceived usefulness by beneficiaries and social norms driven in communities. These differences are essential to the trends and forms the innovation processes take. In this logic of understanding values in innovation processes, we suggest considering innovation in health in the social innovation paradigm: ‘ways of doing things’ [40], social practices [128,129], social relations [130], and transformative and social change [50].

Building on this idea, the narrative review allows us to observe that conditions favourable or unfavourable to scale-up and sustaining health innovation not only depend on the internal innovation environment (innovation and organisational characteristics). These conditions also result from the different elements in the international, national, regional, and local contexts, and more particularly from the relationships between the plurality of actors with different visions, objectives, and values. Therefore, it seems necessary to study these processes to focus on the dynamics of coalitions, alliances, collaborations, negotiations, feedback, and participation implemented in an innovation process, including how they are actualised in organisations and communities and how they influence the continuity and scaling up of health innovations. This knowledge is poorly elucidated in the existing literature. Also, the different temporalities accompanying the entire innovation process are important. They allow us to understand the different events and their sequences and the actions that can lead to successful scaling up and sustainability. Innovation history and its evolution in the contexts of implementation are critical. The various obstacles and barriers in the external environment of innovations for scaling up and sustainability are generally well known. However, research in the literature is lacking on how different elements of innovation and its environment interact and how, over time and space, these elements become risks or opportunities for intervention. On this point, Iwelunmor et al. [2] noted the importance of using systems approaches to better capture the interactions that exist between the components of the intervention and between them and the socio-cultural context of implementation. Noted as well were the organisational and political elements at the broader scale-up of the intervention.

### The proposition of an integrative framework of scale-up and sustainability of social innovation for health

**Figure 3.**
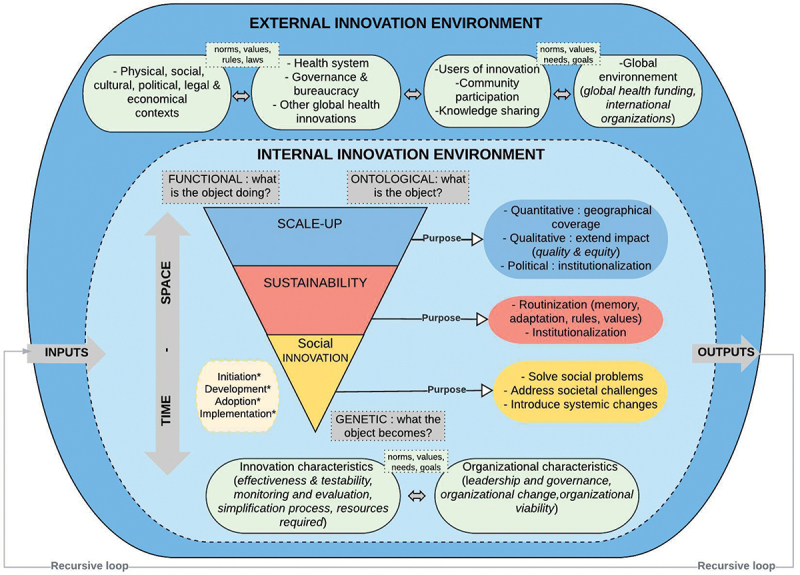
An integrative conceptual framework of scale-up and sustainability.

One of the salient findings of the literature is the lack of a conceptual framework that considers the complexity of scaling-up and sustainability processes. Most existing frameworks propose different stages for sustaining innovation over time and space, and the interactions between contexts (internal and external) are not well elucidated. This gap often leads researchers and practitioners to consider innovation processes separately. In contrast, as shown in this review and other studies, different innovation processes are concomitant and recursive. Recognising this and intending to bridge the gap in research and practice, we propose an integrated conceptual framework for scaling up and sustaining innovation in global health (Figure 3).

The grey colour represents the elements coming from the systems thinking approach presented in the manuscript background. The other boxes represent the different elements found in our literature review. It must be understood that all the elements are interconnected and influence each other. The elements with * presented in the dotted box represent other processes that can take place during the scale-up and sustainability processes.

This framework is derived from our analysis of empirical results of selected documents integrated with a systems thinking approach of innovation. To understand this framework, it must first be considered that it proposes to analyse social innovations in global health as an **open system** with scale-up and sustainability subsystems and other interconnected processes (initiation, development, adoption, implementation). A *sine qua non* of an open system resides in its capacity to transform inputs into outputs while retaining specific criteria, such as its objectives and pursued goals, which can provide information on its success or failure [44]. On this point, the availability of inputs, and prior knowledge of the outputs, to be generated does not guarantee the success of an innovation. For this reason, it is essential to consider the mechanisms and processes of feedback, the **recursive loop** in Figure 3, taking place within and between the innovation and its global environment. It is also simple to determine just the innovation’s internal and external environment factors or determinants; we need to know better the innovation’s capacity for adaptation, stability, learning, and transformation.

The second consideration is **scaling up and sustainability as interconnected processes** in time and space, considering the contexts in which innovation fits and unfolds and the purposes of each process, as the innovation itself, the scale-up and sustainability (Figure 3). Thus, an innovation dynamically interacts at different levels: with itself (between its internal parts or subsystems), with its immediate environment and with the surrounding or super-system environment. Innovation has an inside and outside, and its internal and external environments recursively exchange energy, material, resources, and information. On this point, the internal and external elements of innovation raised in the review results do not exist in a vacuum. These different environments need to be delineated by researchers or practitioners to fully understand the nature of interactions and their impacts on innovation behaviour. On the other hand, it is always necessary to understand how these different environments co-evolve and the impact of this co-evolution on innovation behaviour.

Most of the studies analysed in this review have yet to identify the different levels at which it would be possible to analyse the conditions that do or do not favour scale-up and sustainability. Most of the studies analysed in this review have yet to identify the different levels at which it would be possible to analyse the conditions that do or do not favour scale-up and sustainability. Some studies have identified scalability, but the researcher or practitioner must still learn to analyse innovation behaviours in different contexts and scales. We, therefore, propose that innovation and its subsystems can be observed in terms of: (1) the **functional aspect**, which involves the processes that refer to any transformation or change occurring in time and space [44]. What does innovation do, when, in what environment, and why? (2) The **ontological aspect**, which informs us about the nature of the innovation, its form, and its evolution. What form does the innovation take, taking as its point of reference its conception while following it through different periods, contexts, and scales? (3) The **genetic aspect**, which informs us about its history and future. What history is taking shape during adoption, implementation, scaling up, and sustaining processes – not just the past but also the future; this last point invites us to take a forward-looking view of innovation.

The framework proposed could improve planning for scaling up and sustaining processes that are challenging in practice. Health innovation practitioners could use it to identify the essential elements for sustainability and scaling up innovation, notably by thinking about what is inside or outside the innovation, as developed in the results section. Developed within a global health vision, it has no geographical restrictions for its application. In regard to Northern and Southern settings, they have the same realities about why, how, and when to sustain or scale innovations. For researchers, this framework allows consideration of innovation dynamics while facilitating a systems reading of a set of determinants and processes. Empirical studies should test this framework to properly assess their appropriateness and operationalisation in the global health context. The first author of this manuscript has already used this framework in her empirical study of three global health innovations [14]. The results confirm its usefulness in modelling innovation processes and their contexts. It permits a global vision of innovation studies, delimitating innovation boundaries in time and space and identifying the interconnectedness of critical determinants according to different temporalities of innovation.

## Conclusion

By adopting a systems perspective, this narrative review made it possible to have a different reading of sustainability and scaling up processes. This review suggests going beyond a particular dichotomy of practice and theory to build sustainable knowledge that different experts can use. This not only made it possible to synthesise the existing results in the literature by taking a systematic view of existing publications, but also to deconstruct the idea that innovation is a linear process with a set of characteristics and components. This narrative review concludes that sustainability and scale-up processes are not at the end of the life of an innovation. These processes are a recursive interaction with emergent, evolving properties in time and space and unpredictable, adaptive, and transformative functions. This conclusion was made possible by considering the interactions between innovation processes (conception, adoption, implementation, sustainability, scale-up) and the capacity of transformation through exchanges between inputs and outputs as raised in our framework. The functioning of the innovation in its internal and external environments is an important point. We invite researchers and practitioners to treat these environments as interlocking structures, equilibriums, tensions, and processes interspersed with locally and socially organised actions.

## Paper context

Sustainability and scaling up are studied separately in global health, and few studies address their determinants of success or failure and their interconnected processes. To address this research gap, we conducted this narrative review to get an overview of their success and failure determinants in the social innovations field and proposed an integrated framework based on systems theory. The framework developed could improve research and planning for scaling up and sustaining processes that are challenging in theory and practice.
